# Triazole Resistance and Misidentification of *Aspergillus tubingensis* in Southern California

**DOI:** 10.1001/jamanetworkopen.2025.43630

**Published:** 2025-12-04

**Authors:** Yashan Wang, Maliha Aziz, Kaeley Bush, Gregg S. Davis, Jeffrey T. Foster, Søren Hallstrøm, Amber Jones, Paul S. Keim, Reed Leventis, Cindy M. Liu, Sydney G. Nelson, Daniel E. Park, Magdalena Pomichowski, Vanessa Quinlivan, Emily Rayens, Jason W. Sahl, June Sass, Jessica Skela, Edward Sung, Jack Villani, Matthew Zimmerman, Marc Stegger, Sara Y. Tartof, Lance B. Price

**Affiliations:** 1 Department of Environmental and Occupational Health, Antibiotic Resistance Action Center, The George Washington University, Washington, DC; 2 Department of Research and Evaluation, Kaiser Permanente Southern California, Pasadena; 3 Pathogen and Microbiome Institute, Northern Arizona University, Flagstaff; 4 Department for Sequencing and Bioinformatics, Statens Serum Institut, Copenhagen, Denmark; 5 Department of Global Health, The George Washington University, Washington, DC; 6 Center for Discovery and Innovation, Hackensack Meridian Health, Nutley, New Jersey; 7 Antimicrobial Resistance and Infectious Diseases Laboratory, Harry Butler Institute, Murdoch University, Murdoch, Western Australia, Australia; 8 Kaiser Permanente Southern California and Kaiser Permanente Bernard J Tyson School of Medicine, Pasadena

## Abstract

**Importance:**

Accurate species identification and antifungal susceptibility testing are essential for effective aspergillosis treatment. However, non*–Aspergillus fumigatus *species, such as *Aspergillus tubingensis*, are often misidentified and understudied, potentially compromising proper prognosis and treatment.

**Objective:**

To examine the species and prevalence of triazole resistance among clinical *Aspergillus* isolates in Southern California.

**Design, Setting, and Participants:**

This cross-sectional study collected clinical *Aspergillus* cultures from September 1, 2019, to June 30, 2023, at Kaiser Permanente Southern California, an integrated health system serving a diverse regional population. Triazole susceptibility testing and whole genome sequencing were performed on selected isolates. A total of 2421 consecutive *Aspergillus* cultures were included. Eighty putative *Aspergillus niger* isolates were selected for sequencing, including 44 with positive growth in the presence of at least one clinically relevant triazole.

**Main Outcomes and Measures:**

The primary outcome was the genome-based species identification of *A tubingensis*. The secondary outcome was triazole susceptibility above the *A niger* epidemiological cut-off values. The hypothesis that *A tubingensis* is a prevalent, underrecognized, triazole-resistant pathogen was developed during data collection.

**Results:**

Of 2421 cultures, 1835 were successfully cultured for *Aspergillus*. After purification and deduplication, 1505 isolates were screened for triazole resistance. A substantial fraction of putative *A niger* isolates grew at the *A niger* epidemiological cut-off for itraconazole (110 of 664 [15.1%]). DNA sequencing revealed that 59 of 80 putative *A niger* isolates (73.8%) were actually *A tubingensis*. Elevated triazole minimum inhibitory concentrations were not strongly associated with any known *cyp51* mutations among the *A tubingensis* isolates.

**Conclusions and Relevance:**

In this cross-sectional study of *Aspergillus* isolates from Kaiser Permanente Southern California, *A tubingensis* was a prevalent but underrecognized cause of aspergillosis in Southern California. Its frequent misidentification, association with invasive infections, and triazole resistance underscore the need for improved diagnostics and species-specific epidemiological investigations.

## Key Points

**Question:**

What are the true species identities of *Aspergillus* infections in Southern California, and how common is triazole resistance among them?

**Findings:**

In this cross-sectional study that tested 1505 clinical *Aspergillus* isolates, 664 were identified as *Aspergillus niger* using standard microbiological methods; however, DNA sequencing revealed that 73.8% were actually *Aspergillus tubingensis*. This species showed reduced susceptibility to itraconazole and posaconazole relative to other *Aspergillus* species.

**Meaning:**

*A tubingensis* is a prevalent but largely unrecognized cause of triazole-resistant *Aspergillus* infections in Southern California.

## Teaser

This cross-sectional study assesses species identities and triazole susceptibility among clinical *Aspergillus* isolates in Southern California.

## Introduction

Globally, invasive aspergillosis and chronic pulmonary aspergillosis affect an estimated 4 million people, contributing to 2 million deaths annually, underscoring their considerable impact on global health.^1^ *Aspergillus fumigatus* is currently the dominant species among invasive aspergillosis cases worldwide^2^; however, there has been a marked increase in serious infections caused by the *Aspergillus niger* complex—a group of black spore–forming species in *Aspergillus* section *Nigri*. A retrospective study^3^ in Japan found that respiratory infections due to *A niger* doubled from 2005 to 2009 compared with the previous 5-year period (2000-2004), eventually outnumbering *A fumigatus* as the most frequently isolated species. Similar trends have been observed in Belgium, where invasive infections caused by the *A niger* complex increased 4-fold during the 7 years from 2005 to 2011.^4^

In clinical settings, *Aspergillus* species identification relies primarily on microscopy and culture-based methods, with limited use of matrix-assisted laser desorption/ionization time-of-flight mass spectrometry (MALDI-TOF MS). However, neither method can reliably differentiate among the clinically relevant species that belong to the *A niger* complex.^5,6,7,8^ As a result, these species are often misidentified or reported collectively as *A niger*.^9,10^ *Aspergillus tubingensis*, a frequently misidentified member of this complex, has been increasingly recognized in clinical infections, including respiratory infection and invasive disease in immunocompromised patients.^11,12,13,14^ The inability to accurately differentiate *A tubingensis* from *A niger* may compromise prognosis and treatment.

Triazoles are the first-line treatments for aspergillosis, and the emergence of triazole-resistant *Aspergillus* undermines the effectiveness of these vital medications.^15,16^ Since 2007, triazole-resistant *A fumigatus* has been reported in an increasing number of countries.^17^ In patients with invasive infections, triazole resistance was associated with mortality rates ranging from 62% to 100%.^18,19^ The acquisition of triazole resistance in *A. fumigatus* typically involves alterations in the structure and regulation of the cytochrome P450 sterol 14α-demethylase enzyme encoded by *cyp51A*. Numerous studies^4,5,8,9,10^ have documented triazole resistance in *A tubingensis*, yet its molecular basis remains poorly understood.

In this study, we assessed triazole susceptibility among clinical *Aspergillus* isolates obtained from Kaiser Permanente Southern California (KPSC). Genome sequencing and phylogenetic analysis were performed on a subset of *A niger* complex isolates, revealing a high prevalence of *A tubingensis* among the resistant isolates. To investigate potential resistance mechanisms, we analyzed *cyp51* genes among *A tubingensis* isolates.

## Methods

### Clinical Sample Collection and Fungal Culture

From September 1, 2019, to June 30, 2023, all clinical specimens suspected of *Aspergillus* infection were collected from KPSC, an integrated health system serving more than 4.8 million members across Southern California.^20^ Samples were collected in both inpatient and outpatient settings and processed by the regional microbiology laboratory. Liquid samples were directly plated, whereas solid specimens were macerated before inoculation. Cultures were grown on different media types selected based on specimen source. Cultures were screened within 48 hours and monitored for up to 4 weeks. Initial species identification was based on colony morphologic analysis and microscopy. MALDI-TOF MS (Vitek MS, bioMérieux) was used for confirmation only when pure cultures were available. The study was reviewed and approved by the Kaiser Permanente Southern California institutional review board. We followed all relevant sections of the Strengthening the Reporting of Observational Studies in Epidemiology (STROBE) reporting guideline for cross-sectional studies.

### Clinical *Aspergillus* Isolate Purification

Clinical cultures were transported to The George Washington University for purification and susceptibility testing. *Aspergillus* colonies were streaked onto SabDex agar slants containing chloramphenicol to suppress bacterial growth. Distinct colonies from the same culture were isolated separately. Slants were incubated at 35 °C for 3 to 7 days until sporulation. Growth characteristics and spore colors were recorded, and putative species were assigned to individual isolates based on morphologic findings and clinical laboratory records.

### Triazole Resistance Screening and MIC Testing of *Aspergillus* Isolates

All *Aspergillus* isolates were screened for triazole resistance using the US Centers for Disease Control and Prevention resistance screening method, which uses a quad plate containing itraconazole (4 mg/L), voriconazole (2 mg/L), posaconazole (0.5 mg/L), and a drug-free control.^21^ Growth on any triazole-containing quadrant was interpreted as putative resistance. Isolates that failed screening or were determined to be duplicates were excluded at this step (eFigure 1 in Supplement 1). Minimum inhibitory concentration (MIC) testing (Liofilchem) was performed on all putative resistant isolates and a subset of susceptible *A fumigatus* isolates and *A niger* complex isolates. Resistance was defined using species- and method-specific epidemiological cut-off values for *A niger* (in the absence of epidemiological cut-offs for *A tubingensis*): 4 mg/L for itraconazole, 1 mg/L for voriconazole (different from screening concentration), and 0.5 mg/L for posaconazole.^22^ Additional MIC testing for isavuconazole was conducted on all sequenced *A niger* complex isolates with epidemiological cut-offs at 4 mg/L for isavuconazole. Agreement between screening and MIC test strips was evaluated on a subset of isolates (eMethods 2 in Supplement 1).

### Triazole Treatment History of Patients With Resistant *Aspergillus*

Previous antifungal treatment in patients with triazole-resistant *Aspergillus* infection was assessed using KPSC electronic health records, including both laboratory testing and pharmacy records. Care received outside KPSC was incorporated through claims reimbursement. Patient records were reviewed for azole prescriptions (posaconazole, itraconazole, isavuconazole, voriconazole, or fluconazole) within the year before culture collection, incorporating pharmacy data from both inpatient and outpatient encounters.

### *A niger* Complex Isolate Selection and Whole Genome Sequencing

Forty-four *A niger* complex strains with positive growth in the presence of any of the 3 triazoles (itraconazole, voriconazole, or posaconazole) were matched with 36 strains that did not grow on triazole-containing quadrants; matching variables included specimen type, collection date, and patient zip code. Isolates were cultured for 1 to 3 days to produce a hyphal mat, from which 50 to 200 mg was harvested for DNA extraction using a modified protocol (DNeasy Plant Mini protocol, Qiagen). Mechanical lysis was performed with tungsten and glass beads using a sample homogenizer (TissueLyser II, Qiagen) for 1 minute. DNA quality and quantity were assessed using gel electrophoresis and Qubit fluorometry.

DNA samples were sequenced to generate 2 × 100-bp paired-end reads (NextSeq 500 or NovaSeq 6000, Illumina). Resultant short reads were quality checked using FastQC, version 0.11.8 (Babraham Institute) and assembled using SPAdes genome assembler, version 3.15.5^23^ and then quality checked with QUAST, version 2.3.^24^ Additional details on library preparation and reagents used are provided in eMethods 1 in Supplement 1.

### Genome Curation and Phylogenetic Analysis

Publicly available *Aspergillus* genomes, including whole genomes and mitochondrial genomes, were curated from the Joint Genome Institute and National Center for Biotechnology Information (NCBI) databases (eTable 1 in Supplement 1). Nomenclature follows the taxonomy proposed by Houbraken et al.^25^ Phylogenetic trees were constructed using single-nucleotide polymorphisms derived from the core genome or mitochondrial genome. Short reads were mapped to the *A niger* mitochondrial genome (GenBank accession No. NC_007445) or *A tubingensis* whole genome (GenBank accession No. KV878176-KV878208) using the NASP pipeline, version 1.2.0^26^ with BWA-mem, version 0.7.12.^27^ Single-nucleotide polymorphisms were called using GATK, version 4.2.4.0, and sites affected by presence-absence variation were reintegrated at a 25% threshold. Recombinant regions were filtered with Gubbins, version 3.3.1,^28^ and maximum-likelihood trees were constructed in PhyML, version 3.3 with 100 bootstraps. Metadata was annotated in iTOL, version 6.^29^ The Consistency Index was calculated by Homoplasy Finder.^30^ The NeighborNet phylogenetic network and Delta score were generated using SplitsTree, version 6.^31^

### Mating Type and *cyp51* Gene Analysis

*A tubingensis* assemblies were screened for the MAT1-1 (GenBank accession No. KC848774), MAT1-2 (GenBank accession No. KC848776), *cyp51A *(GenBank accession No. MH781351), and *cyp51B* (GenBank accession No. XM_035504231) genes using BLAST+, version 2.12.0 (National Center for Biotechnology Information). For *cyp51* genes, sequences were aligned in Geneious Prime, version 2024.0 (Geneious). A susceptible strain consensus was generated to represent the wild type. Sequences of *cyp51A* from triazole-resistant strains were compared with the reference and consensus. Tandem repeats (pattern size ≥10 bp and identity ≥80%) within 3000 bp upstream or downstream of the *cyp51A* gene were detected using Tandem Repeats Finder.^32^

## Results

### *Aspergillus* Species and Variation in Clinical Samples

From September 1, 2019, to June 30, 2023, a total of 2421 *Aspergillus* cultures were identified at KPSC. Most (1910 [78.9%]) cultures contained a single *Aspergillus* species, whereas the remainder involved multiple *Aspergillus* species or coinfections with other fungi. Among all cultures, the top species identified by the clinical laboratory were *A niger* (n = 1002), *A fumigatus* (n = 996), and *Aspergillus terreus* (n = 316) (Table 1). Species varied by specimen source: 798 *A. fumigatus* cultures (80.1%) came from respiratory samples compared with 448 (44.7%) for *A niger* (Table 1). We successfully purified *Aspergillus* isolates from 1835 of 2421 clinical samples (75.8%), resulting in 1917 isolates, of which 1505 isolates received putative species assignments and underwent further analysis (eFigure 1 in Supplement 1). In this step, all isolates initially reported as *A niger* by the clinical laboratory were reclassified under the *A niger* complex to reflect taxonomic uncertainty before molecular identification.

**Table 1.** Clinical Specimen Sources of Top *Aspergillus* Species

Specimen types	No. (%) of specimens		
	*Aspergillus niger* ^a^	*Aspergillus fumigatus*	*Aspergillus terreus*
Respiratory specimens			
Sputum	199 (19.9)	313 (31.4)	42 (13.3)
Bronchial lavage	165 (16.5)	290 (29.1)	30 (9.5)
Bronchial wash	68 (6.8)	120 (12.0)	9 (2.8)
Nasal	14 (1.4)	54 (5.4)	14 (4.4)
Lung	2 (0.2)	11 (1.1)	1 (0.3)
Other respiratory	0	10 (1.0)	5 (1.6)
Nonrespiratory specimens			
Nail	177 (17.7)	47 (4.7)	194 (61.4)
Skin	169 (16.9)	56 (5.6)	12 (3.8)
Fluid	77 (7.7)	27 (2.7)	2 (0.6)
Wound	73 (7.3)	16 (1.6)	4 (1.3)
Tissue	17 (1.7)	25 (2.5)	0
Other nonrespiratory	41 (4.1)	27 (3.7)	3 (0.9)

^a^ This species designation is from clinical laboratory records based on morphologic testing and mass spectrometry. DNA sequencing indicated that many of the isolates initially identified as *Aspergillus niger* were actually *Aspergillus tubingensis*.

### Prevalence of Triazole Resistance Among Clinical *Aspergillus* Isolates

On the basis of the Centers for Disease Control and Prevention triazole resistance screening method, *A niger* complex showed the highest rate of putative resistance among all major species, with more than 15.1% growing in the presence of itraconazole at the *A niger* epidemiological cut-offs (Table 2). However, MIC testing of a subset of *A fumigatus* and *A niger* complex isolates showed only moderate agreement between MICs and plate-based screening, with voriconazole showing the strongest agreement (83.8%-88.5%) followed by itraconazole (55.0%-80.8%) and posaconazole (51.3%-69.2%) (eTables 2 and 3 in Supplement 1). Clinical records showed that 12 of 91 resistant *A niger* complex isolates (13.2%) and 3 of 12 resistant *A fumigatus* isolates (25.0%) were from patients with a history of azole treatment in the previous year.

**Table 2.** Triazole Resistance Screening for Major Clinical *Aspergillus* Species Based on the US Centers for Disease Control and Prevention Screening Method

Species^a^	Total isolates screened	No. (%) of isolates			
		Positive growth with any triazoles^b^	Positive growth with itraconazole	Positive growth with voriconazole	Positive growth with posaconazole
*Aspergillus niger* complex^c^	664	105 (15.8)	100 (15.1)	6 (0.9)	5 (0.8)
*Aspergillus* fumigatus	531	12 (2.3)	9 (1.7)	7 (1.3)	4 (0.8)
*Aspergillus terreus*	201	4 (2.0)	3 (1.5)	2 (1.0)	0
*Aspergillus flavus/oryzae*	37	3 (8.1)	1 (2.7)	3 (8.1)	1 (2.7)
*Aspergillus nidulans*	22	3 (13.6)	3 (13.6)	0	0
*Aspergillus lentulus*	20	4 (20.0)	3 (15.0)	2 (10.0)	0
*Aspergillus sydowii*	19	3 (15.8)	3 (15.8)	0	0
*Aspergillus versicolor*	5	0	0	0	0
Other	6	3 (50.0)	1 (16.7)	1 (16.7)	3 (50.0)
Total	1505	137 (9.1)	123 (8.2)	21 (1.4)	13 (0.9)

^a^ Only species with 5 or more isolates screened are included. Species are determined using clinical diagnosis or based on morphologic testing at the purification step.

^b^ Susceptibility testing using minimal inhibitory concentrations suggests that this standard screening method originally developed for *Aspergillus fumigatus* is not optimized for screening *Aspergillus tubingensis* (eTables 2 and 3 in Supplement 1).

^c^ Isolates initially reported as *Aspergillus niger* by the clinical laboratory were reclassified under the *A niger* complex to reflect taxonomic uncertainty.

### Species Composition of *A niger* Complex

**Figure 1.**
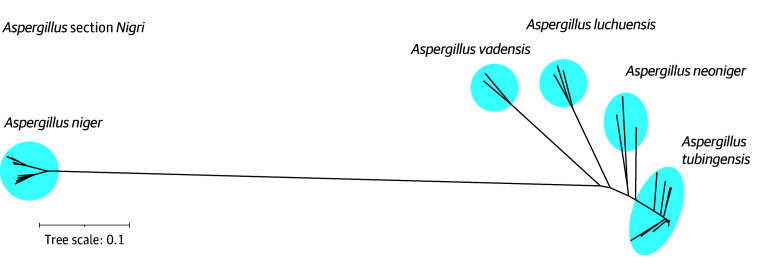
Core-Genome Phylogeny of *Aspergillus* Section Nigri Based on 97 Isolates, of Which 80 Were From Kaiser Permanente Southern California (KPSC) This unrooted tree, based on 5 277 451 core single-nucleotide polymorphisms, was constructed using an *Aspergillus tubingensis* genome as a reference and added genomes from the National Center for Biotechnology Information as context (see eTable 1 in Supplement 1).

Phylogenetic analysis revealed that *A tubingensis* was the most common species among *A niger* complex clinical isolates. The genus-level phylogeny (eFigure 2 in Supplement 1), based on mitochondrial genes, confirmed that all 80 sequenced black-spored clinical isolates belonged to *Aspergillus* section *Nigri* series *Nigri*. Core-genome phylogeny of section *Nigri* (Figure 1) further revealed that *A tubingensis* was the most prevalent species among clinical isolates (59 [73.8%]), followed by *A niger* (12 [15.0%]), *Aspergillus luchuensis* (4 [5.0%]), *Aspergillus vadensis* (4 [5.0%]), and *Aspergillus neoniger* (1 [1.2%]). Current and former species names are provided in eTable 4 in Supplement 1.

### Triazole MICs Among Sequencing-Confirmed *A tubingensis* Isolates

We determined MICs for itraconazole, voriconazole, posaconazole, and isavuconazole in all sequenced *A niger* complex isolates (eTable 3 in Supplement 1). Among the 59 *A tubingensis* isolates, 42 (71.2%) had MICs above the *A niger* epidemiological cut-offs. To balance our genomic analysis, resistant and susceptible isolates had been sequenced in approximately equal numbers based on initial screening results. Elevated MICs were most frequent for posaconazole (n = 32) followed by itraconazole (n = 25), voriconazole (n = 11), and isavuconazole (n = 4). Seventeen isolates showed elevated MICs to both itraconazole and posaconazole, and one had elevated MICs to all 4 drugs. MIC ranges were 0.5 to greater than 32 mg/L for itraconazole, 0.047 to 3 mg/L for voriconazole, 0.19 to 1.5 mg/L for posaconazole, and 0.25 to 8 mg/L for isavuconazole (eTable 3 in Supplement 1).

### Analysis of *A tubingensis cyp51* Paralogs

**Figure 2.**
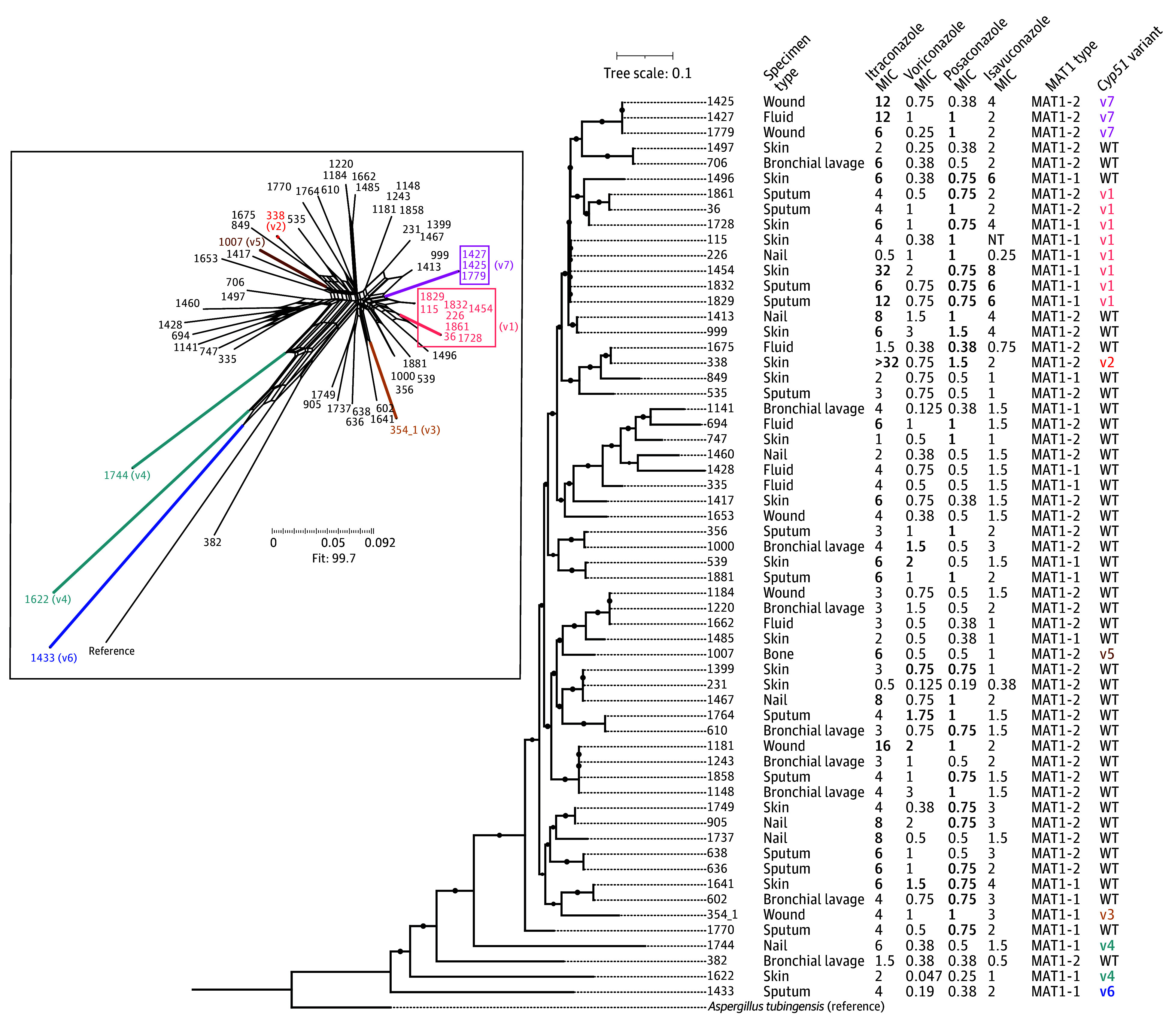
Core-Genome Phylogenetic Tree and Phylogenetic Network of *Aspergillus tubingensis* A total of 1 112 898 core single-nucleotide polymorphisms were called among the 60 *Aspergillus tubingensis* isolates of 80 *Aspergillus niger* complex isolates. Isolates 1829 and 1832 as well as 636 and 638 are 2 pairs that came from the same patients, but samples were collected on different dates. The tree is annotated with specimen type, triazole minimum inhibitory concentrations (MICs), MAT1 type, and *cyp51* gene variant patterns defined in Table 3. The inserted figure is the NeighborNet phylogenetic network of *A tubingensis* colored by *cyp51* gene variants. v1 to v5 are mutations in *cyp51A*; v6, mutations in *cyp51A* and *cypB*; and v7, mutations in *cyp51B*. NT indicates not tested; WT, wild type.

We analyzed the *cyp51A* and *cyp51B* paralogs in the *A tubingensis* isolates for potential associations with variation in triazole susceptibility. The *cyp51A* gene was usually 1595 bp, spanning 2 exons, and *cyp51B* was 1061 bp. In 12 of the 59 *A tubingensis* isolates (20.3%), we identified a *cyp51A* intronic insertion, sometimes co-occurring with specific amino acid changes within the downstream exon (Table 3). Across all isolates, we observed 7 total distinct missense mutation patterns, with 6 involving *cyp51A* and 2 involving *cyp51B*, as annotated on the *A tubingensis* phylogeny (Figure 2). Although promoter repeats are known to be a common resistance mechanism in *A fumigatus*, no tandem repeats were found within 3000 bp upstream or downstream of *cyp51A* in our sample population, indicating a difference in resistance mechanisms among species.

**Table 3.** Variants of *cyp51* Genes and Amino Acid Substitutions in Sequenced *Aspergillus tubingensis* Isolates

*cyp51* variant	No. (with azole history) of strains^a^	*cyp51A* intronic insertion	*cyp51A* amino acid substitutions	*cyp51B* amino acid substitutions	MIC for variants ≥3 isolates, minimum/median/maximum, mg/L			
					Itraconazole	Voriconazole	Posaconazole	Isavuconazole
WT	42 (8)	No	None	None	0.5/4/16	0.13/0.75/3	0.19/0.75/1.5	0.38/1.5/6
v1	8 (2)	Yes	A9V, L21F, T321A, I503V	None	0.5/5/32	0.38/0.88/2	0.75/0.75/1	0.25/4/8
v2	1 (0)	No	M218I	None	>32	0.75	1.5	2
v3	1 (0)	No	L4F, A185G	None	4	1	1	3
v4	2 (0)	Yes	V17A	None	2/6	0.047/0.38	0.25/0.5	1/1.5
v5	1 (0)	No	G509E	None	6	0.5	0.5	1
v6	1 (0)	No	E32K, T321A	I325M	4	0.19	0.38	2
v7	3 (1)	No	None	A32V	6/12/12	0.25/0.75/1	0.38/1/1	2/2/4

Abbreviations: MIC, minimum inhibitory concentration; WT, wild type.

^a^ Number of isolates of that variant and the number of isolates that had an azole prescription in the past year.

The most common *cyp51A* variant (1596 bp, n = 8) carried the substitutions A9V, L21F, T321A, and I503V, with an intronic insertion at position 211. Four of these isolates exhibited elevated MICs to both itraconazole and posaconazole, including one that had elevated MICs for all 4 drugs. A M218I substitution was found exclusively in a highly resistant isolate (>32 mg/L for itraconazole and 1.5 mg/L for posaconazole). In *cyp51B*, the A32V substitution appeared in 3 isolates resistant to itraconazole. Nevertheless, 6 isolates that had MICs above the *A niger* epidemiological cut-offs for itraconazole, voriconazole, and posaconazole had wild-type *cyp51* sequences. eFigure 3 in Supplement 1 maps the *A tubingensis* amino acid substitutions and intron indels alongside well-characterized triazole resistance–associated *cyp51A* mutations in *A fumigatus* extracted from AFRbase.^33^

### Phylogenetic Distribution of Triazole Susceptibility and *cyp51A* Variants in *A tubingensis*

Core-genome phylogenetic analysis of the *A tubingensis* isolates showed that there was some phylogenetic clustering of *cyp51A* variants but no consistent patterns associated with triazole susceptibility (Figure 2). Isolates with triazole MICs above the *A niger* epidemiological cut-offs were distributed throughout the tree and were not limited to either mating type (MAT1-1 or MAT1-2). The NeighborNet phylogenetic network (Figure 2), which may better represent sexually reproducing populations, showed a similar clustering of *cyp51A* variants. Additional population genetics of *A tubingensis* can be found in the eResults in Supplement 1.

## Discussion

Our study revealed that nearly three-quarters of clinical isolates initially identified as *A niger* based on morphologic findings were in fact *A tubingensis* on molecular characterization, highlighting this cryptic species as an underrecognized cause of aspergillosis in Southern California. This finding illustrates a broader challenge in clinical microbiology: routine laboratory methods often fail to differentiate cryptic species within the *A niger* complex. Even genome-based approaches are imperfect; a recent large-scale phylogenomic study^34^ found that nearly 8% of publicly available *Aspergillus* genomes were misidentified. Such diagnostic limitations may obscure important trends in disease burden, resistance rates, and clinical outcomes.

*A tubingensis* represented a substantial contributor to the overall burden of aspergillosis in Southern California. With the *A niger* complex being the most frequently isolated group during the study period and *A tubingensis* the predominant species within the group, it likely accounted for more than a quarter of all *Aspergillus* isolates. More than one-third of *A tubingensis* isolates were recovered from respiratory specimens, suggesting a potential role in invasive disease. Although *A fumigatus* remains the predominant cause of respiratory aspergillosis, our findings highlight *A tubingensis* as a clinically relevant but underrecognized respiratory pathogen. Further research is needed to clarify its virulence relative to other common *Aspergillus* species and to inform management strategies.

Triazole antifungals are essential for treating *Aspergillus* infections, but the emergence of resistant strains threatens this paradigm. Species that are intrinsically less susceptible to triazoles further complicate management. Our in vitro data suggest that itraconazole and posaconazole have limited clinical utility against *A tubingensis*, whereas resistance to voriconazole and isavuconazole was less common, supporting current treatment guidelines.^15,35^ The limited treatment options underscore the importance of antifungal stewardship across clinical and environmental domains.

Agricultural triazole fungicides share structural similarity with medical triazoles and can select for resistance in environmental fungi.^36,37^ In California, nearly 200 000 kg of triazoles are applied annually to orchards, vineyards, and other commodities.^38^ Because *A tubingensis* is a soil-dwelling saprophyte, such exposure could select for strains with reduced susceptibility or increase the species’ environmental abundance. This represents a potential pathway in which agricultural fungicide use expands environmental reservoirs of resistance, increasing the risk of human exposure to resistant *A tubingensis*.^39^

The genetic basis of variation in triazole susceptibility in *A tubingensis* remains unresolved. We identified several *cyp51A* mutations, including a previously reported variant pattern (A9V, L21F, T321A, I503V) linked to reduced triazole susceptibility, but no consistent associations with MICs or phylogeny were observed.^5,10^ Other genetic factors may also influence triazole susceptibility in *A tubingensis*.^40,41^ In other *Aspergillus* species, overexpression of efflux transporters (eg, *cdr1B* in *A fumigatus*) reduces intracellular azole concentrations.^42,43^ Mutations in genes encoding regulatory transcription factors, such as *AtrR* and *HapE*, as well as enzymes such as Hmg1, have also been linked to resistance through altered drug-target expression or ergosterol biosynthesis.^44,45^ In addition, mitochondrial dysfunction and calcium signaling influence azole susceptibility in *Candida* species, although their roles in *Aspergillus* remain less defined. Broader transcriptomic and functional studies are needed to determine whether these pathways contribute to variation in *A tubingensis* isolates lacking known *cyp51* alterations. Finally, technical variation inherent to MIC testing may also explain some of the phenotypic diversity observed in this study, underscoring the need for *A tubingensis*–specific methods and reference data to reduce uncertainty.

### Strengths and Limitations

Our study has several important strengths. By leveraging a large and diverse collection of clinical samples gathered during 4 years, we were able to characterize *A tubingensis* as an underrecognized fungal pathogen. We addressed a critical gap by conducting the first large-scale US analysis of the *A niger* complex and resolving cryptic species identity with genomic methods. Furthermore, we used genomic data to explore potential molecular contributors to variation in triazole susceptibility. Collectively, these findings enhance understanding of an overlooked fungal pathogen and provide a foundation for future surveillance, diagnostic, and treatment strategies.

This study also has some limitations. A key limitation was the lack of practical clinical tools to differentiate species within the *A niger* complex, which necessitated reliance on genomic sequencing. Consequently, our *A tubingensis* analysis was limited to isolates with genome sequence data. We were also constrained by the absence of standardized screening protocols and epidemiological cut-offs for *A tubingensis.* To estimate resistance prevalence, we applied a screening protocol developed for *A fumigatus* and interpreted results using *A niger* epidemiological cut-offs, introducing uncertainty. Without *A tubingensis*–specific epidemiological cut-offs, we could not determine whether the elevated MICs observed in some strains fell outside the species’ wild-type distribution, and we could not distinguish between intrinsic and acquired resistance mechanisms. Finally, because our study was conducted within a single health system in Southern California, the generalizability of these findings to other regions remains uncertain.

## Conclusions

In this cross-sectional study of *Aspergillus* isolates from KPSC, *A tubingensis* constituted a significant proportion of isolates and made up most cultures previously classified as *A niger*. This finding suggests that the burden of disease caused by *A tubingensis* has been underappreciated, likely due to longstanding challenges in accurately identifying species within the *A niger* complex. Further research is warranted to elucidate the mechanisms of triazole resistance in *A tubingensis*, the potential role of agricultural fungicides in shaping environmental reservoirs, and whether *A tubingensis* represents an emerging threat or a long-overlooked pathogen beyond Southern California.

## Notes

Supplement 1. **eMethods 1.** Short-read sequencing and assembly **eMethods 2.** Comparison of agreement between screening and MIC Test Strips **eTable 1.** MIC Test Strips results for *A. fumigatus* (n = 26) and agreement with the CDC triazole resistance screening **eTable 2.** MIC Test Strips results for *A. niger* complex (n = 80) and agreement with the CDC triazole resistance screening **eTable 3.** Key synonyms for species in *Aspergillus* section *Nigri* **eTable 4.** Reference genomes and other external genomes used in the phylogenetic analysis **eResults.** Population structure of *A. tubingensis* **eFigure 1.** Clinical *Aspergillus* isolates workflow. **eFigure 2.** Mitochondrial DNA phylogeny of clinically-relevant *Aspergillus *species **eFigure 3.** Comparative mapping of *cyp51A* amino acid substitutions and intron indels, including associated triazole resistance mutations, in *Aspergillus tubingensis* and *A. fumigatus*
eReferences

Supplement 2.
Data Sharing Statement
