# Supplementary material for: Triazole Resistance and Misidentification of Aspergillus tubingensis in Southern California
Source: JAMA Netw Open. 2025 Dec 4;8(12):e2543630. doi: 10.1001/jamanetworkopen.2025.43630 (PMC12679326; doi:10.1001/jamanetworkopen.2025.43630)
Supplement: Supplement 1. — eMethods 1. Short-read sequencing and assembly eMethods 2. Comparison of agreement between screening and MIC Test Strips eTable 1. MIC Test Strips results for A. fumigatus (n = 26) and agreement with the CDC triazole resistance screening eTable 2. MIC Test Strips results for A. niger complex (n = 80) and agreement with the CDC triazole resistance screening eTable 3. Key synonyms for species in Aspergillus section Nigri eTable 4. Reference genomes and other external genomes used in the phylogenetic analysis eResults. Population structure of A. tubingensis eFigure 1. Clinical Aspergillus isolates workflow. eFigure 2. Mitochondrial DNA phylogeny of clinically-relevant Aspergillus species eFigure 3. Comparative mapping of cyp51A amino acid substitutions and intron indels, including associated triazole resistance mutations, in Aspergillus tubingensis and A. fumigatus eReferences [file jamanetwopen-e2543630-s001.pdf]

Supplemental Online Content

Wang Y, Aziz M, Bush K, et al. Triazole resistance and misidentification of *Aspergillus tubingensis* in Southern California. *JAMA Netw Open or JAMA Health Forum*. 2025;8(11):e2543630. doi:10.1001/jamanetworkopen.2025.43630

eMethods 1. Short-read sequencing and assembly

eMethods 2. Comparison of agreement between screening and MIC Test Strips

eTable 1. Reference genomes and other external genomes used in the phylogenetic analysis.

eTable 2. MIC Test Strips results for *A. fumigatus* (n = 26) and agreement with the CDC triazole resistance screening

eTable 3. MIC Test Strips results for *A. niger* complex (n = 80) and agreement with the CDC triazole resistance screening

eTable 4. Key synonyms for species in *Aspergillus* section *Nigri*

eResults. Population structure of *A. tubingensis*

eFigure 1. Clinical *Aspergillus* isolates workflow.

eFigure 2. Mitochondrial DNA phylogeny of clinically-relevant *Aspergillus* species.

eFigure 3. Comparative mapping of *cyp51A* amino acid substitutions and intron indels, including associated triazole resistance mutations, in *Aspergillus tubingensis* and *A. fumigatus*.

eReferences

This supplemental material has been provided by the authors to give readers additional information about their work.

© 2025 Wang Y et al. *JAMA Network Open*.

eMethods 1. Short-read sequencing and assembly

Sequencing was performed in two batches using a NextSeq 500 instrument or a NovaSeq 6000 instrument. For the NextSeq run, DNA libraries were constructed using the KAPA HyperPrep Kit and KAPA Unique Dual-Indexed Adapter Kit (Roche, Switzerland), with double-sided size selection. Libraries were quantified using a QuantStudio 7 Flex Real-Time PCR System (Applied Biosystems, USA) and pooled at equimolar concentrations. Sequencing was performed using the High Output KT v2.5 (300 cycles) Reagent Kit following Illumina protocols. For the NovaSeq run, the sequencing libraries were made using a custom-modified version of Illumina's DNA Prep, pooled at equimolar ratios, and sequenced using an S4 Reagent Kit v1.5 (200 cycles) producing 2x100 bp paired-end sequencing reads.

Illumina sequencing of 80 *Aspergillus* isolates yielded a total 4,700 million reads, all had a Phred quality score ≥ Q30. The reads were assembled into an average of 2,000 contigs per isolate.

eMethods 2. Comparison of agreement between screening and MIC Test Strips

To verify that the U.S. Centers for Disease Control and Prevention (CDC) triazole resistance screening method had comparable accuracy for *Aspergillus fumigatu*s and *Aspergillus niger* complex, Minimum Inhibitory Concentration (MIC) testing was performed on 12 putative resistant isolates and 14 randomly-selected susceptible *A. fumigatus* isolates for itraconazole (ITC), voriconazole (VOR) and posaconazole (POS) (eTable 2). MIC testing was also performed on all sequenced *A. niger* complex, which included 44 putative resistant and 36 susceptible isolates for ITC, VOR, POS, and isavuconazole (IVU) (eTable 3).

© 2025 Wang Y et al. *JAMA Network Open*.

eTable 1. Reference genomes and other external whole genomes (WGS) or mitochondrial genome (Mt) used in the phylogenetic analysis.

Label Name	Accession	Genome Source	Label Name	Accession	Genome Source
A. tubingensis whole genome Reference	KV878176-KV878208	WGS_NCBI	A. niger-15	GCA_023134235.1	WGS_NCBI
A. niger mitochondrial DNA Reference	NC_007445	Mt_NCBI	A. niger-16	GCA_023134265.1	WGS_NCBI
A. tubingensis MAT1-1	KC848774	NCBI	A. niger-17	GCA_023134295.1	WGS_NCBI
A. tubingensis MAT1-2	KC848776	NCBI	A. niger-18	GCA_023134325.1	WGS_NCBI
A. tubingensis cyp51A Reference	MH781351	NCBI	A. niger-19	GCA_023134355.1	WGS_NCBI
A. tubingensis cyp51B Reference	XM_035504231	RefSeq_NCBI	A. niger-20	GCA_023134385.1	WGS_NCBI
A. brasiliensis-1	GCA_037892995.1	WGS_NCBI	A. niger-21	GCA_023134415.1	WGS_NCBI
A. flavus-1	CM022151	Mt_NCBI	A. niger-22	GCA_023134445.1	WGS_NCBI
A. flavus-2	CM022153	Mt_NCBI	A. niger-23	GCA_023134475.1	WGS_NCBI
A. flavus-3	CM022154	Mt_NCBI	A. niger-24	GCA_023134515.1	WGS_NCBI
A. flavus-4	CM023206	Mt_NCBI	A. niger-25	GCA_023137675.1	WGS_NCBI
A. flavus-5	CM023208	Mt_NCBI	A. niger-26	GCA_023137705.1	WGS_NCBI
A. flavus-6	CM023210	Mt_NCBI	A. niger-27	GCA_023137735.1	WGS_NCBI
A. flavus-7	CM023216	Mt_NCBI	A. niger-28	GCA_023137765.1	WGS_NCBI
A. flavus-8	CM023218	Mt_NCBI	A. niger-29	GCA_023137795.1	WGS_NCBI
A. flavus-9	GCA_003967795.1	WGS_NCBI	A. niger-30	GCA_023137805.1	WGS_NCBI
A. flavus-10	GCA_013436355.1	WGS_NCBI	A. niger-31	GCA_023137835.1	WGS_NCBI
A. flavus-11	GCA_014784225.2	WGS_NCBI	A. niger-32	GCA_023137865.1	WGS_NCBI
A. flavus-12	GCA_031304255.1	WGS_NCBI	A. niger-33	GCA_023137895.1	WGS_NCBI

© 2025 Wang Y et al. *JAMA Network Open*.

Label Name	Accession	Genome Source	Label Name	Accession	Genome Source
A. fumigatus-1	CM016889	Mt_NCBI	A. niger-34	GCA_023137925.1	WGS_NCBI
A. fumigatus-2	GCA_012656115.1	WGS_NCBI	A. niger-35	GCA_023137955.1	WGS_NCBI
A. fumigatus-3	GCA_012656125.1	WGS_NCBI	A. niger-36	GCA_023137965.1	WGS_NCBI
A. fumigatus-4	GCA_012656165.1	WGS_NCBI	A. niger-37	GCA_023618375.1	WGS_NCBI
A. fumigatus-5	GCA_012656185.1	WGS_NCBI	A. niger-38	GCA_023625355.1	WGS_NCBI
A. fumigatus-6	GCA_012656215.1	WGS_NCBI	A. niger-39	GCA_024862975.1	WGS_NCBI
A. fumigatus-7	GCA_023620365.1	WGS_NCBI	A. niger-40	GCA_025768975.1	WGS_NCBI
A. fumigatus-8	GCA_029618165.1	WGS_NCBI	A. niger-41	GCA_025769215.1	WGS_NCBI
A. fumigatus-9	GCA_029618175.1	WGS_NCBI	A. niger-42	GCA_025769265.1	WGS_NCBI
A. fumigatus-10	GCA_029618185.1	WGS_NCBI	A. niger-43	GCA_025769395.1	WGS_NCBI
A. fumigatus-11	GCA_029618195.1	WGS_NCBI	A. niger-44	GCA_026119785.1	WGS_NCBI
A. fumigatus-12	GCA_029618205.1	WGS_NCBI	A. niger-45	GCA_027923725.1	WGS_NCBI
A. fumigatus-13	GCA_029618225.1	WGS_NCBI	A. niger-46	GCA_027923745.1	WGS_NCBI
A. fumigatus-14	GCA_029618255.1	WGS_NCBI	A. niger-47	GCA_027923785.1	WGS_NCBI
A. fumigatus-15	GCA_029618275.1	WGS_NCBI	A. niger-48	GCA_027923805.1	WGS_NCBI
A. fumigatus-16	GCA_029618285.1	WGS_NCBI	A. niger-49	GCA_027923825.1	WGS_NCBI
A. fumigatus-17	GCA_029618305.1	WGS_NCBI	A. niger-50	GCA_027923865.1	WGS_NCBI
A. fumigatus-18	GCA_029618335.1	WGS_NCBI	A. niger-51	GCA_027923945.1	WGS_NCBI
A. fumigatus-19	GCA_040126065.1	WGS_NCBI	A. niger-52	GCA_027923985.1	WGS_NCBI
A. fumigatus-20	GCA_040142845.1	WGS_NCBI	A. niger-53	GCA_027924005.1	WGS_NCBI
A. fumigatus-21	GCA_040142865.1	WGS_NCBI	A. niger-54	GCA_029783925.1	WGS_NCBI
A. fumigatus-22	GCA_040142965.1	WGS_NCBI	A. niger-55	GCA_900248155.1	WGS_NCBI
A. lentulus-1	GCA_010723975.1	WGS_NCBI	A. niger-56	GCF_003184595.1	WGS_NCBI
A. lentulus-2	GCA_010724575.1	WGS_NCBI	A. ochraceus-1	GCA_004849945.1	WGS_NCBI
A. lentulus-3	GCA_010724635.1	WGS_NCBI	A. ochraceus-2	GCA_005784425.1	WGS_NCBI
A. lentulus-4	GCA_010724915.1	WGS_NCBI	A. ochraceus-3	GCA_026108165.1	WGS_NCBI

© 2025 Wang Y et al. *JAMA Network Open*.

Label Name	Accession	Genome Source	Label Name	Accession	Genome Source
A. lentulus-5	GCA_012656335.1	WGS_NCBI	A. oryzae-1	GCF_000184455.2	WGS_NCBI
A. lentulus-6	GCA_012656375.1	WGS_NCBI	A. oryzae-2	GCA_000269785.2	WGS_NCBI
A. lentulus-7	GCA_012656395.1	WGS_NCBI	A. oryzae-3	GCA_000691885.1	WGS_NCBI
A. lentulus-8	GCA_012656435.1	WGS_NCBI	A. oryzae-4	NC_008282	Mt_NCBI
A. lentulus-9	GCA_012656445.1	WGS_NCBI	A. sydowii-1	GCA_009193685.1	WGS_NCBI
A. lentulus-10	GCA_012656455.1	WGS_NCBI	A. sydowii-2	GCA_040113305.1	WGS_NCBI
A. lentulus-11	GCF_010724455.1	WGS_NCBI	A. terreus-1	GCF_000149615.1	WGS_NCBI
A. luchuensis-1	GCF_016861625.1	WGS_NCBI	A. terreus-2	GCA_002749855.1	WGS_NCBI
A. luchuensis-2	GCA_000239835.2	WGS_NCBI	A. terreus-3	GCA_009014675.2	WGS_NCBI
A. luchuensis-3	GCA_001602395.1	WGS_NCBI	A. terreus-4	GCA_009932835.1	WGS_NCBI
A. luchuensis-4	GCA_001890685.1	WGS_NCBI	A. terreus-5	GCA_016808415.1	WGS_NCBI
A. luchuensis-5	GCA_016860305.1	WGS_NCBI	A. terreus-6	GCA_023625495.1	WGS_NCBI
A. luchuensis-6	GCA_016860325.1	WGS_NCBI	A. terreus-7	GCA_023625515.1	WGS_NCBI
A. luchuensis-7	GCA_016860345.1	WGS_NCBI	A. terreus-8	GCA_033632065.1	WGS_NCBI
A. luchuensis-8	GCA_016860365.1	WGS_NCBI	A. tubingensis-1	DQ217399	Mt_NCBI
A. luchuensis-9	GCA_016860385.1	WGS_NCBI	A. tubingensis-2	GCA_001890745.1	WGS_NCBI
A. luchuensis-10	GCA_016860405.1	WGS_NCBI	A. tubingensis-3	GCA_010614855.1	WGS_NCBI
A. luchuensis-11	GCA_016860425.1	WGS_NCBI	A. tubingensis-4	GCA_019805365.1	WGS_NCBI
A. luchuensis-12	GCA_016860445.1	WGS_NCBI	A. tubingensis-5	GCA_019827425.1	WGS_NCBI
A. luchuensis-13	GCA_016865315.1	WGS_NCBI	A. tubingensis-6	GCA_019827445.1	WGS_NCBI
A. luchuensis-14	GCA_037074665.1	WGS_NCBI	A. tubingensis-7	GCA_019827465.1	WGS_NCBI
A. luchuensis-15	MK061298	Mt_NCBI	A. tubingensis-8	GCA_019827565.1	WGS_NCBI

© 2025 Wang Y et al. *JAMA Network Open*.

Label Name	Accession	Genome Source	Label Name	Accession	Genome Source
A. luchuensis-16	NC_040166	Mt_NCBI	A. tubingensis-9	GCA_019828785.1	WGS_NCBI
A. neoniger-1	GCA_029783945.1	WGS_NCBI	A. tubingensis-10	GCA_027923605.1	WGS_NCBI
A. neoniger-2	GCF_003184625.1	WGS_NCBI	A. tubingensis-11	GCA_027923625.1	WGS_NCBI
A. nidulans-1	GCF_000149205.2	WGS_NCBI	A. tubingensis-12	GCA_027923665.1	WGS_NCBI
A. nidulans-2	GCA_011074995.1	WGS_NCBI	A. tubingensis-13	GCA_027923685.1	WGS_NCBI
A. nidulans-3	GCA_011075025.1	WGS_NCBI	A. tubingensis-14	GCA_027923885.1	WGS_NCBI
A. nidulans-4	GCA_025766005.1	WGS_NCBI	A. tubingensis-15	GCA_027923925.1	WGS_NCBI
A. nidulans-5	NC_017896	Mt_NCBI	A. tubingensis-16	GCA_030762845.1	WGS_NCBI
A. niger-1	GCF_000002855.4	WGS_NCBI	A. tubingensis-17	GCA_040333235.1	WGS_NCBI
A. niger-2	Aspni_NRRL3_1	WGS_JGI	A. tubingensis-18	GCA_900163765.1	WGS_NCBI
A. niger-3	GCA_000230395.2	WGS_NCBI	A. tubingensis-19	GCF_013340325.1	WGS_NCBI
A. niger-4	CM041301	Mt_NCBI	A. tubingensis-20	NC_007597	Mt_NCBI
A. niger-5	CM041305	Mt_NCBI	A. vadensis-1	GCF_003184925.1	WGS_NCBI
A. niger-6	GCA_000633045.1	WGS_NCBI	A. versicolor-1	GCA_020284045.1	WGS_NCBI
A. niger-7	GCA_001515345.1	WGS_NCBI	A. versicolor-2	GCA_037043815.1	WGS_NCBI
A. niger-8	GCA_002211485.2	WGS_NCBI	A. welwitschiae-1	GCA_009761105.1	WGS_NCBI
A. niger-9	GCA_003344705.1	WGS_NCBI	A. welwitschiae-2	GCA_012275225.1	WGS_NCBI
A. niger-10	GCA_004634315.1	WGS_NCBI	A. welwitschiae-3	GCA_012365075.1	WGS_NCBI

© 2025 Wang Y et al. *JAMA Network Open*.

Label Name	Accession	Genome Source	Label Name	Accession	Genome Source
A. niger-11	GCA_015586235.1	WGS_NCBI	A. welwitschiae-4	GCA_023718355.1	WGS_NCBI
A. niger-12	GCA_019288275.1	WGS_NCBI	A. welwitschiae-5	GCA_030015405.1	WGS_NCBI
A. niger-13	GCA_023091205.1	WGS_NCBI	A. welwitschiae-6	GCA_037044315.1	WGS_NCBI
A. niger-14	GCA_023091265.1	WGS_NCBI			

© 2025 Wang Y et al. *JAMA Network Open*.

eTable 2. MIC Test Strips results for *A. fumigatus* (n = 26) and agreement with the CDC triazole resistance screening

Isolate ID	ITC-screening	ITC-MIC	VOR-screening	VOR-MIC	POS-screening	POS-MIC
5	N	0.75	N	0.094	N	0.064
23	Y	1.5	N	0.25	N	0.25
56	N	1	N	0.125	N	0.19
131	N	0.25	Y	32	N	0.03
227	Y	4	Y	2	Y	3
242	N	1.5	N	0.19	N	0.25
542	Y	1	Y	0.13	Y	0.25
571	N	1.5	N	0.19	N	0.19
622	N	1.5	N	0.19	N	0.38
661	Y	2	Y	0.25	Y	0.19
769	N	2	Y	32	N	1
795	N	0.75	N	0.125	N	0.125
827	N	1	N	0.19	N	0.125
872	N	1.5	Y	0.13	N	0.13
940	N	1	N	0.19	N	0.19
1061	N	1	N	0.125	N	0.19
1132	N	1	N	0.094	N	0.19
1152	Y	12	Y	3	N	1
1229	Y	0.75	N	0.125	N	0.19
1443	Y	>32	N	0.047	Y	12
1526	N	1	N	0.125	N	0.19
1571	N	0.75	N	0.125	N	0.25
1915	Y	1	N	0.5	N	0.75
1938	N	1.5	N	0.19	N	0.25
1963	Y	>32	N	0.023	N	3
2130	N	1.5	N	0.19	N	0.38

© 2025 Wang Y et al. *JAMA Network Open*.

eTable 3. MIC Test Strips results for *A. niger* complex (n = 80) and agreement with the CDC triazole resistance screening

Isolate ID	Species	ITC-screening	ITC-MIC	VOR-screening	VOR-MIC	POS-screening	POS-MIC	IVU-MIC*
29	*A. luchuensis*	N	6	N	1	N	0.75	3
36	*A. tubingensis*	Y	4	N	1	N	1	2
112	*A. luchuensis*	N	1.5	N	0.5	N	0.38	1.5
115	*A. tubingensis*	Y	4	N	0.38	N	1	NT[#]
226	*A. tubingensis*	N	0.5	N	1	Y	1	0.25
231	*A. tubingensis*	N	0.5	N	0.125	N	0.19	0.38
335	*A. tubingensis*	N	4	N	0.5	N	0.5	1.5
338	*A. tubingensis*	Y	>32	N	0.75	N	1.5	2
354 1	*A. tubingensis*	Y	4	N	1	N	1	3
356	*A. tubingensis*	Y	3	N	1	N	1	2
382	*A. tubingensis*	N	1.5	N	0.38	N	0.38	0.5
390	*A. niger*	N	2	N	0.38	N	0.38	1.5
535	*A. tubingensis*	N	3	N	0.75	N	0.5	1
539	*A. tubingensis*	Y	6	N	2	N	0.5	1.5
585	*A. neoniger*	N	3	N	0.5	N	0.25	0.75
602	*A. tubingensis*	Y	4	N	0.75	N	0.75	3
610	*A. tubingensis*	Y	3	N	0.75	N	0.75	1.5
613	*A. niger*	N	2	N	0.25	N	0.38	0.75
636	*A. tubingensis*	N	6	N	1	N	0.75	2
638	*A. tubingensis*	Y	6	N	1	N	0.5	3
693	*A. niger*	N	6	Y	>32	N	1.5	1.5
694	*A. tubingensis*	N	6	N	1	N	1	1.5
705	*A. niger*	Y	8	Y	>32	N	2	3
706	*A. tubingensis*	N	6	N	0.38	N	0.5	2
747	*A. tubingensis*	Y	1	N	0.5	N	1	1

© 2025 Wang Y et al. *JAMA Network Open*.

Isolate ID	Species	ITC-screening	ITC-MIC	VOR-screening	VOR-MIC	POS-screening	POS-MIC	IVU-MIC*
754	*A. vadensis*	N	4	N	0.5	N	0.5	2
843	*A. niger*	N	1.5	N	0.125	N	0.38	1
849	*A. tubingensis*	N	2	N	0.75	Y	0.5	1
895	*A. luchuensis*	N	4	N	0.75	N	0.5	NT#
905	*A. tubingensis*	Y	8	N	2	N	0.75	3
987	*A. luchuensis*	Y	4	N	1	N	0.38	1
989	*A. niger*	N	8	Y	32	N	0.75	2
999	*A. tubingensis*	N	6	N	3	N	1.5	4
1000	*A. tubingensis*	N	4	N	1.5	N	0.5	3
1002	*A. vadensis*	N	3	N	0.75	N	0.75	1.5
1007	*A. tubingensis*	N	6	Y	0.75	N	0.75	1
1141	*A. tubingensis*	Y	4	N	0.125	N	0.38	1.5
1148	*A. tubingensis*	N	4	N	3	N	1	1.5
1181	*A. tubingensis*	N	16	N	2	N	1	2
1184	*A. tubingensis*	Y	3	N	0.75	N	0.5	1.5
1220	*A. tubingensis*	Y	3	N	1.5	N	0.5	2
1243	*A. tubingensis*	N	3	N	1	N	0.5	2
1399	*A. tubingensis*	N	3	N	0.75	N	0.75	1
1413	*A. tubingensis*	N	8	N	1.5	N	1	4
1417	*A. tubingensis*	Y	6	N	0.75	N	0.38	1.5
1422	*A. vadensis*	Y	3	N	0.38	N	0.38	1
1425	*A. tubingensis*	N	12	N	0.75	N	0.38	4
1427	*A. tubingensis*	Y	12	N	1	N	1	2
1428	*A. tubingensis*	Y	4	N	0.75	N	0.5	1.5
1433	*A. tubingensis*	Y	4	N	0.19	N	0.38	2
1446	*A. niger*	N	3	N	0.38	N	0.75	1.5
1449	*A. vadensis*	N	3	N	0.38	N	0.38	1.5

© 2025 Wang Y et al. *JAMA Network Open*.

Isolate ID	Species	ITC-screening	ITC-MIC	VOR-screening	VOR-MIC	POS-screening	POS-MIC	IVU-MIC*
1454	*A. tubingensis*	Y	32	N	2	N	0.75	8
1460	*A. tubingensis*	N	2	N	0.38	N	0.5	1.5
1467	*A. tubingensis*	Y	8	N	0.75	N	1	2
1485	*A. tubingensis*	Y	2	N	0.5	N	0.38	1
1496	*A. tubingensis*	N	6	N	0.38	N	0.75	6
1497	*A. tubingensis*	N	2	N	0.25	N	0.38	2
1622	*A. tubingensis*	N	2	N	0.047	N	0.25	1
1641	*A. tubingensis*	Y	6	N	1.5	N	0.75	4
1653	*A. tubingensis*	N	4	N	0.38	N	0.5	1.5
1662	*A. tubingensis*	N	3	N	0.5	N	0.38	1
1675	*A. tubingensis*	Y	1.5	N	0.38	N	0.38	0.75
1728	*A. tubingensis*	Y	6	N	1	N	0.75	4
1737	*A. tubingensis*	N	8	N	0.5	N	0.5	1.5
1744	*A. tubingensis*	Y	6	N	0.38	N	0.5	1.5
1749	*A. tubingensis*	N	4	N	0.38	N	0.75	3
1764	*A. tubingensis*	Y	4	N	1.75	N	1	1.5
1770	*A. tubingensis*	Y	4	N	0.5	N	0.75	2
1779	*A. tubingensis*	Y	6	N	0.25	N	1	2
1809	*A. niger*	N	2	N	0.125	N	0.38	1
1817	*A. niger*	Y	3	N	0.75	N	0.75	1.5
1829	*A. tubingensis*	Y	12	Y	0.75	N	0.75	6
1832	*A. tubingensis*	Y	6	N	0.75	N	0.75	6
1834	*A. niger*	Y	2	N	0.19	N	0.38	1
1835	*A. niger*	Y	2	N	0.25	N	0.5	1
1858	*A. tubingensis*	Y	4	N	1	N	0.75	1.5
1861	*A. tubingensis*	Y	4	N	0.5	N	0.75	2
1881	*A. tubingensis*	Y	6	N	1	N	1	2

© 2025 Wang Y et al. *JAMA Network Open*.

Isolate ID	Species	ITC-screening	ITC-MIC	VOR-screening	VOR-MIC	POS-screening	POS-MIC	IVU-MIC*
1892	*A. niger*	N	2	N	0.19	N	0.25	0.75

*Screening was not performed with IVU.
#Additional MIC test strip for IVU was performed after the initial test and two isolates 115 and 895 were not tested (NT) due to lost viability.

© 2025 Wang Y et al. *JAMA Network Open*.

eTable 4. Key synonyms for species in *Aspergillus* section *Nigri.* Nomenclature follows the taxonomy proposed by Houbraken et al.[1]

**Current Name**	**Deprecated Synonyms (Selected)**
Aspergillus luchuensis	*A. awamori, A. acidus, A. pulverulentus*
Aspergillus niger	*A. awamori* (in part), *A. foetidus, A. phoenicis, A. ficuum, A. lacticoffeatus*
Aspergillus tubingensis	*A. niger var. tubingensis, A. quercinus* (in part)

Population structure of *A. tubingensis*

The *A. tubingensis* core-genome phylogeny (Figure 2) comprised 1.1 million SNPs (mean pairwise distance: 136,763 SNPs; range: 9–358,744). The average Consistency Index for the parsimony tree was 0.73. Both mating types were present in this *A. tubingensis* population, supporting the potential for sexual recombination in this species. We subsequently assessed recombination using a NeighborNet network, which showed a Delta score of 0.34, suggesting a moderate level of reticulate evolution (i.e., occasional mating and exchange of genetic material between strains); however, the distribution of certain *cyp51* mutations and mating types also suggested a history of limited clonal expansions.

© 2025 Wang Y et al. *JAMA Network Open*.

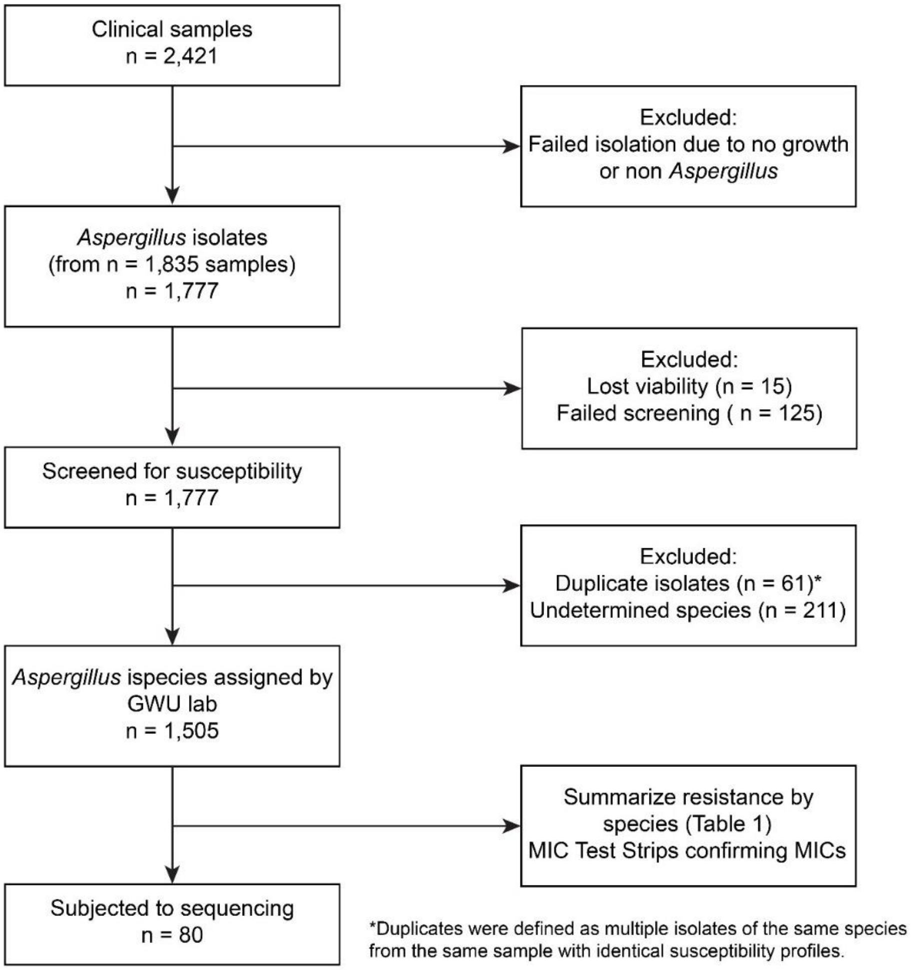

eFigure 1. Clinical *Aspergillus* isolates workflow.

© 2025 Wang Y et al. *JAMA Network Open*.

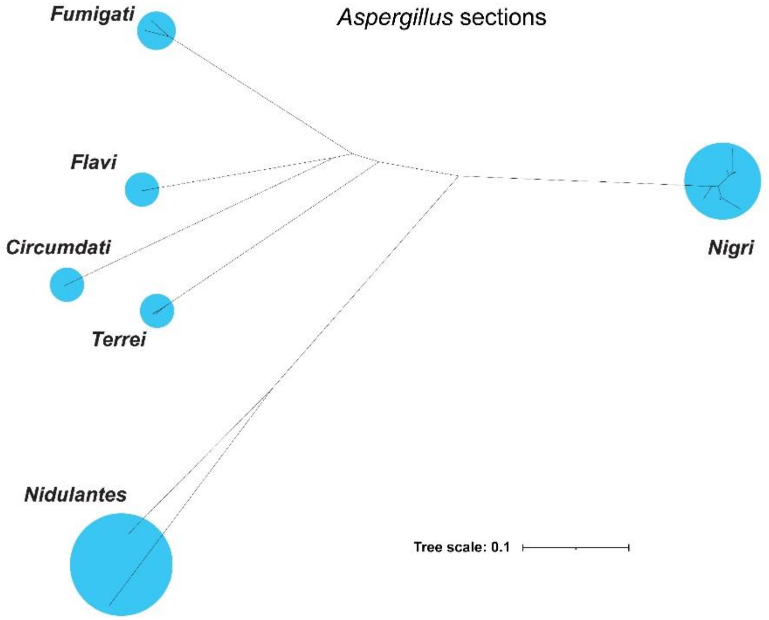

eFigure 2. Mitochondrial DNA phylogeny of clinically-relevant *Aspergillus* species. A total of 4,378 core Single Nucleotide Polymorphisms (SNPs) were identified using an *A. niger* mitochondrial DNA as a reference (NCBI Accession No: NC_007445). All sequences from Kaiser Permanente Southern California (n = 80) located in the *Aspergillus* section *Nigri* clade.

© 2025 Wang Y et al. *JAMA Network Open*.

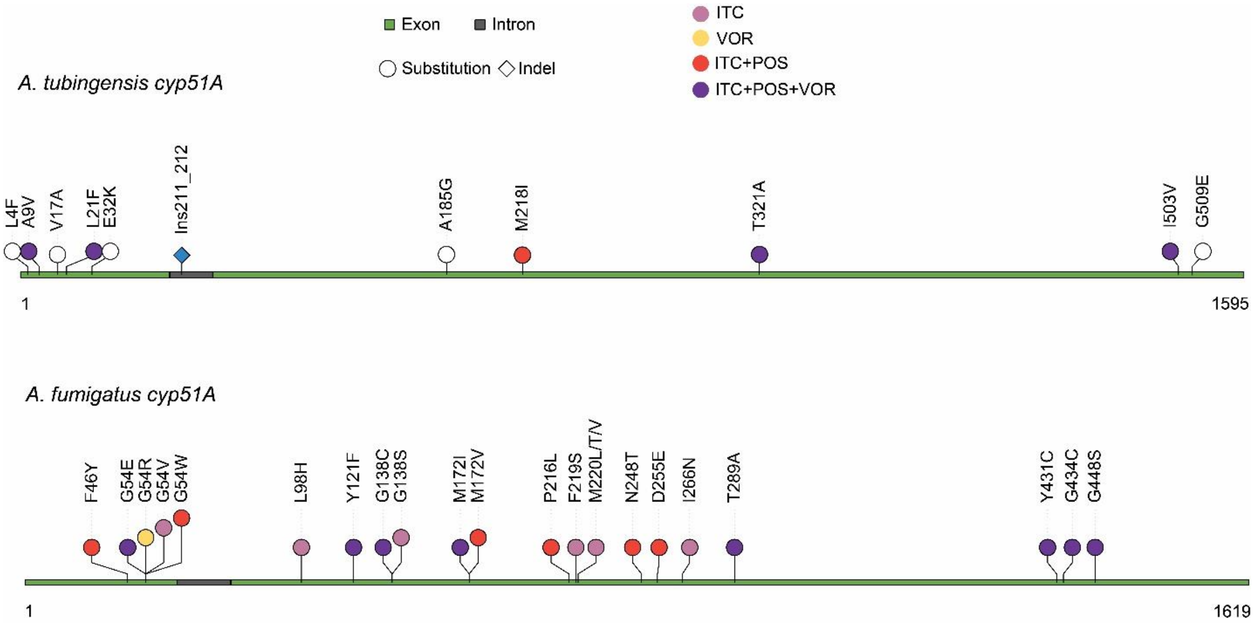

eFigure 3. Comparative mapping of *cyp51A* amino acid substitutions and intron indels, including associated triazole resistance mutations, in *Aspergillus tubingensis* and *A. fumigatus*. Top panel: amino acid substitutions and intron indels identified in *A. tubingensis* isolates in this study. Bottom panel: well-characterized triazole resistance-associated *cyp51A* mutations in *A. fumigatus*, extracted from AFRbase.[2]

eReferences

1. Houbraken J, Kocsubé S, Visagie CM, et al. Classification of Aspergillus, Penicillium, Talaromyces and related genera (Eurotiales): An overview of families, genera, subgenera, sections, series and species. *Stud Mycol*. 2020;95:5-169.

2. Jain A, Singhal N, Kumar M. AFRbase: a database of protein mutations responsible for antifungal resistance. *Bioinformatics*. 2023;39(11). doi:10.1093/bioinformatics/btad677

© 2025 Wang Y et al. *JAMA Network Open*.
