# Supplementary material for: Triazole Resistance and Misidentification of Aspergillus tubingensis in Southern California
Source: JAMA Netw Open. 2025 Dec 4;8(12):e2543630. doi: 10.1001/jamanetworkopen.2025.43630 (PMC12679326; doi:10.1001/jamanetworkopen.2025.43630)
Supplement: Supplement 2. — Data Sharing Statement [file jamanetwopen-e2543630-s002.pdf]

Data Sharing Statement

Wang. Triazole Resistance and Misidentification of Aspergillus tubingensis in Southern California. *JAMA Netw Open*. Published December 05, 2025.
doi:10.1001/jamanetworkopen.2025.43630

Data
Data available: Yes
Data types: Deidentified participant data, Data (not involving human participants)
How to access data: The genomes generated in this study were submitted to the NCBI under BioProject ID PRJNA1253755. Patient data that support the findings of this study may be made available from the investigative team in the following conditions: (1) agreement to collaborate with the study team on all publications, (2) provision of external funding for administrative and investigator time necessary for this collaboration, (3) demonstration that the external investigative team is qualified and has documented evidence of training for human subjects protections, and (4) agreement to abide by the terms outlined in data use agreements between institutions. Please email to sara.y.tartof@kp.org
When available: With publication

Supporting Documents
Document types: None

Additional Information
Who can access the data: Qualified investigators who agree to the terms above may request access.
Types of analyses: For specific purposes.
Mechanisms of data availability: Access is provided through institutional data use agreements and requires collaboration with the study team as outlined above.
